# Cardiac tumours in children

**DOI:** 10.1186/1750-1172-2-11

**Published:** 2007-03-01

**Authors:** Orhan Uzun, Dirk G Wilson, Gordon M Vujanic, Jonathan M Parsons, Joseph V De Giovanni

**Affiliations:** 1 Consultant Paediatric Cardiologist, Department of Paediatric Cardiology, University Hospital Of Wales, Heath Park Cardiff, CF14 4XW, Wales, UK; 2 Consultant Senior Lecturer in Paediatric Pathology, School of Medicine, Cardiff University, Heath Park, Cardiff CF14 4XW, Wales, UK; 3 Consultant Paediatric Cardiologist, Yorkshire Heart Centre, Department Of Paediatric Cardiology, Leeds, UK; 4 Consultant Paediatric Cardiologist. Birmingham Children's Hospital, Birmingham, UK

## Abstract

Cardiac tumours are benign or malignant neoplasms arising primarily in the inner lining, muscle layer, or the surrounding pericardium of the heart. They can be primary or metastatic. Primary cardiac tumours are rare in paediatric practice with a prevalence of 0.0017 to 0.28 in autopsy series. In contrast, the incidence of cardiac tumours during foetal life has been reported to be approximately 0.14%. The vast majority of primary cardiac tumours in children are benign, whilst approximately 10% are malignant. Secondary malignant tumours are 10–20 times more prevalent than primary malignant tumours. Rhabdomyoma is the most common cardiac tumour during foetal life and childhood. It accounts for more than 60% of all primary cardiac tumours. The frequency and type of cardiac tumours in adults differ from those in children with 75% being benign and 25% being malignant. Myxomas are the most common primary tumours in adults constituting 40% of benign tumours. Sarcomas make up 75% of malignant cardiac masses. Echocardiography, Computing Tomography (CT) and Magnetic Resonance Imaging (MRI) of the heart are the main non-invasive diagnostic tools. Cardiac catheterisation is seldom necessary. Tumour biopsy with histological assessment remains the gold standard for confirmation of the diagnosis. Surgical resection of primary cardiac tumours should be considered to relieve symptoms and mechanical obstruction to blood flow. The outcome of surgical resection in symptomatic, non-myxomatous benign cardiac tumours is favourable. Patients with primary cardiac malignancies may benefit from palliative surgery but this approach should not be recommended for patients with metastatic cardiac tumours. Surgery, chemotherapy and radiotherapy may prolong survival. The prognosis for malignant primary cardiac tumours is generally extremely poor.

## Definition, epidemiology and diagnosis

Cardiac tumours are benign or malignant neoplasms arising primarily in the inner lining, muscle layer, or the surrounding pericardium of the heart. Cardiac tumours can be primary or metastatic. Primary cardiac tumours are rare in paediatric practice with a prevalence of 0.0017 to 0.28 in autopsy series. In contrast, the incidence of cardiac tumours during fetal life has been reported to be approximately 0.14% [1-3].

The vast majority of primary cardiac tumours in children are benign, whilst approximately 10% are malignant. Secondary malignant tumours are 10–20 times more prevalent than primary malignant tumours [4]. The frequency and type of cardiac tumours in adults differ from children with 75% being benign and 25% being malignant. Myxomas are the most common primary tumours in adults constituting 40% of benign tumours. Sarcomas make up 75% of malignant cardiac masses [5,6].

Rhabdomyoma is the most common cardiac tumour during fetal life and childhood (Table 1). This is usually followed by teratoma, fibroma and haemangioma [3]. Myxoma is exceedingly rare in foetuses and neonates [4,7]. Likewise, fibroma and intracardiac teratoma are exceptionally rare in adults but more commonly seen in the first year of life during childhood. Rhabdomyoma and pericardial teratoma make up more than 70% of the primary tumours of the heart in foetuses, neonates, and infants [8,9].

**Table 1:** Frequency Distribution of Cardiac Tumours in Children

*Primary Benign*	*Frequency (%)*
Rhabdomyoma	40-60
Teratoma	15-19
Fibroma	12-16
Myxoma	2-4
Haemangioma	5
Lymphangioma	}
Haemangiopericytoma	} Very rate
Oncocytic tumours	}
	
***Primary Malignant***	
Rabdomyosarcomas	2
Fibrosarcoma	2
	
***Secondary Metastatic tumours***	
Neuroblastoma	}
Leukaemia	} very
Lymphoma	} rare
Melanoma	}

**Figure 1:**
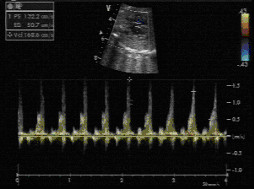
A foetal tumour in the left ventricular (LV) cavity causing LV outflow tract obstruction.

**Figure 2:**
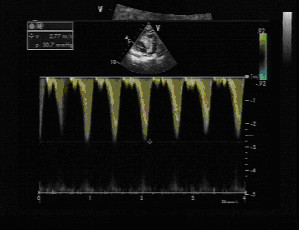
Right ventricular (RV) tumour in a 2-year-old child resulting in RV outflow tract obstruction. The Doppler velocity is 2.77 m/sec.

Cardiac tumours may present in foetal or post-natal life. The presenting features depend on the size and location of the mass. In the foetus a tumour can be noted on a routine antenatal anomaly scan as an intracardiac mass. The manifestations of a cardiac tumour in foetal life include arrhythmia, congestive heart failure, hydrops, and not infrequently stillbirth. In postnatal life cardiac tumours may affect the integrity and function of the adjacent cardiac structures leading to severely compromised blood flow due to inflow (Figure 1) or outflow tract obstruction (Figure 2), cyanosis, murmur, respiratory distress, myocardial dysfunction, valvular insufficiency, arrhythmias, and sudden death [2,8].

The diagnosis of cardiac tumours can be established in symptomatic patients, but in rare cases sudden death is the presenting feature. Echocardiography or Magnetic Resonance Imaging (MRI) is usually adequate to facilitate the diagnosis of cardiac tumours. Cardiac catheterisation is rarely necessary. Tumour biopsy, with histological assessment, remains as the gold standard for confirmation of the diagnosis.

**Figure 3:**
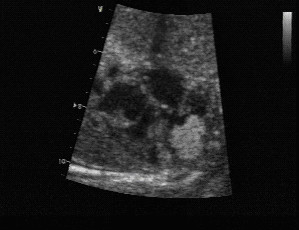
Multiple rhabdomyomas are found on a foetal ultrasound at 20 weeks of gestation. Rhabdomyomas appears brighter than the surrounding myocardium.

Due to the progressive nature of pregnancy, fetal cardiac tumours are expected to grow antenatally and it is not unusual for cardiac lesions to be missed at an early obstetric scan. Some tumours can be detected from 20 weeks onwards but the majority will develop later in the course of the pregnancy (Figure 3). Most fetal cardiac tumours will be readily detectable in the late second or third trimester [3,8].

## Benign cardiac tumours

### Rhabdomyoma

The most common primary cardiac tumour in infants and children is rhabdomyoma. It accounts for more than 60% of all primary cardiac tumours [6,7]. Rhabdomyomas are usually located within the ventricles but not infrequently they may also originate in the atriums. Their location in the atrioventricular junction may have a rather peculiar effect where tumour may act like an accessory pathway with resultant pre-excitation on twelve lead electrocardiogram (ECG).

Fetal diagnosis of cardiac rhabdomyoma is most commonly made on a 20-week anomaly scan after incidental detection of multiple intracardiac masses. Occasionally, cardiac rhabdomyoma may be noted coincidentally during evaluation for fetal cardiac arrhythmia.

Although the association of multiple cardiac rhabdomyomas with tuberous sclerosis has long been recognised, the association with a single rhabdomyoma is not clear. However, in case of a solitary tumour a careful examination of cardiac chambers should be made in order not to miss smaller lesions elsewhere. Multiple cardiac rhabdomyomas in fetal life may herald the diagnosis of tuberous sclerosis well before the other features of the disease, such as skin signs or seizures, emerge in infancy [10,11]. The incidence of tuberous sclerosis in patients with cardiac rhabdomyoma has been reported to be between 60–80% [12,13].

Equally in patients with the diagnosis of tuberous sclerosis, cardiac rhabdomyoma can be detected between 43–72% of cases and their presence may assist in making a definitive diagnosis of the condition [10,11]. However, the diagnosis of tuberous sclerosis must only be made on the basis of strict criteria, and the presence of multiple cardiac rhabdomyomas, whilst raising strong suspicions, is not by itself sufficient evidence to establish the diagnosis. Nevertheless, detection of multiple cardiac tumours should raise a strong suspicion of rhabdomyoma, hence tuberous sclerosis. In such patients a detailed family history should be obtained and genetic counselling should be offered [14].

**Figure 4:**
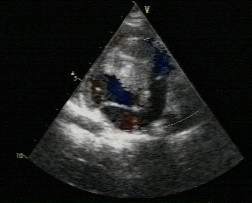
A tumour in the RV cavity results in RV midcavity obstruction.

**Figure 5:**
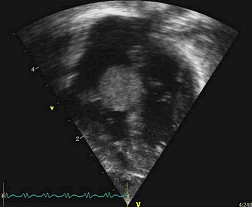
Rhabdomyoma on the tricuspid valve with some inflow obstruction in a different patient.

After birth, symptoms are commonly related to obstruction of inflow or outflow tracts (Figures 4 and 5).

**Figure 6:**
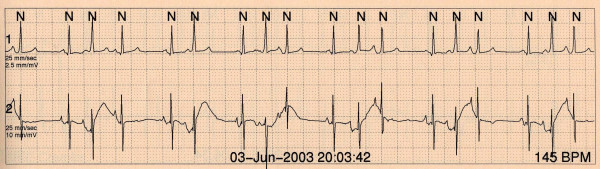
A 24-hour Holter on a child a month after birth shows pre-excitation and premature atrial beats.

**Figure 7:**
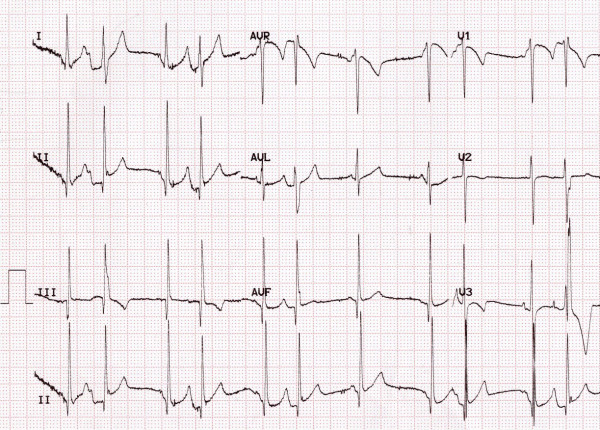
In the same child aged 3 years the 12-lead ECG reveals loss of pre-excitation but ongoing premature atrial complexes.

Clinical examination may reveal a murmur, reduced peripheral pulses or cyanosis. Arrhythmias are not a rare event and have been reported to occur in 16 to 47% of cases with cardiac rhabdomyoma. Atrial or ventricular arrhythmias frequently occur but a higher incidence of Wolff-Parkinson-White syndrome has been noted in patients with tuberous sclerosis (1.5%) compared to the general population (0.15%). This may be explained by the fact that the tumour cells may create continuity at the atrioventricular junction between the atrial and ventricular myocardium. Since the tumour cells are structurally similar to Purkinje cells they can function like accessory pathways [15]. As the tumour regresses, the associated arrhythmias also undergo spontaneous resolution and loss of the delta wave is frequently encountered [16] (Figures 6 and 7).

**Figure 8:**
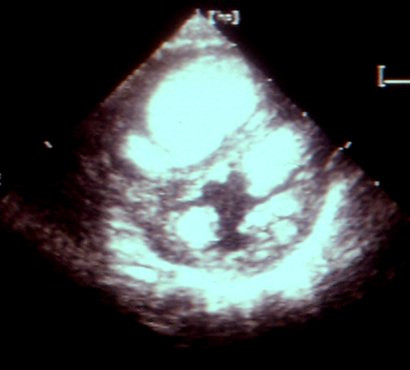
Echocardiography on a newborn child shows multiple rhabdomyomas in both ventricles. In this child the diagnosis of tuberous sclerosis was confirmed.

Macroscopically rhabdomyomas appear uniform, round and solid, and are brighter than the neighbouring healthy myocardium. Their predominant location is the ventricular myocardium, but they can also be seen in the atrial wall. Rhabdomyomas are most commonly multiple and intracavitary extensions are found in up to 50% of patients. Echocardiography is diagnostic and shows multiple bright intramural masses with luminal extensions (Figure 8).

Microscopically each tumour shows pathognomonic spider cells with centrally placed cytoplasm containing the nucleus and myofibrils radiating to the cell wall. Rhabdomyomas are not considered to be true tumours and many authors would describe them as hamartomas of striated muscular fibres occurring solely in the heart [17]. Rhabdomyomas exhibit immunoreactivity with the muscle markers desmin, actin, myoglobin, vimentin, and also with hamartin and tuberin. Immunohistochemical studies showed that the spider cells exhibit immunoreactivity with ubiquitin. The ubiquitin pathway is associated with the degradation of myoflaments, progression of cytoplasmic vacuolisation with the formation of spider cells, enlargement of glycogen vacuoles, apoptosis, myxoid degeneration and regression of the rhabdomyomas. These above events may provide plausible explanation for the spontaneous regression of rhabdomyomas [18]. The outcome of antenatally detected cardiac rhabdomyomas is favourable. Once foetal somatic growth is completed, hamartomas lose their mitotic potential and undergo apoptosis [19]. The majority of tumours will regress towards the end of the third trimester although rarely some may continue to grow larger. Despite the expected shrinkage of these tumours, unexpected foetal loss may occur due to arrhythmias or obstruction of blood flow.

After birth, rhabdomyoma cells lose their ability to divide and regression of the tumour in infancy is an expected outcome, regardless of size of the tumour [20-22]. Hence, following birth, tumour regression is a rule rather than an exception. Complete resolution of more than 80% of the tumours may occur during early childhood [23].

Thus, due to high rate of spontaneous regression, after birth these tumours can be managed conservatively with echocardiography and ECG monitoring. Surgical intervention should be preserved only for sick patients with symptoms of severe obstruction with haemodynamic compromise or haemodynamically significant and intractable arrhythmias that are unresponsive to antiarrhythmic drugs [24,25].

### Fibroma

Fibromas are solitary tumours that are mainly located in the ventricular septum. Fibromas are derived from connective tissue fibroblasts. They are primarily seen in childhood and the size may vary from 1 to 10 cm. Fibroma may invade the ventricular muscle, replace the working myocardium and may result in intractable congestive heart failure or cyanosis. Not infrequently, a fibroma may extend into the ventricular conduction system and can cause ventricular arrhythmias [26-28].

**Figure 9:**
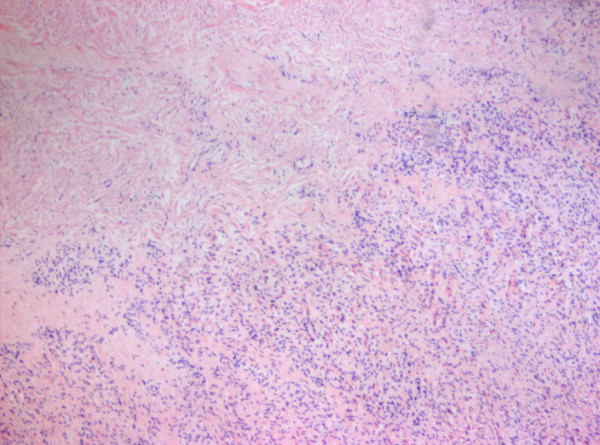
Fibroma of the heart of a foetus. There is macerated myocardium.

**Figure 10:**
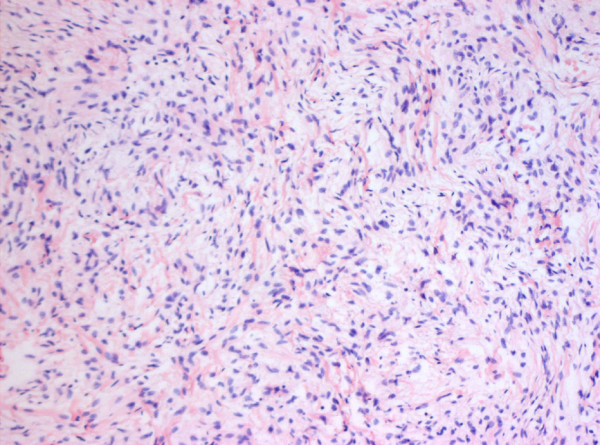
The second image shows that the tumour composed of fibroblasts arranged in fascicles.

Macroscopically, a fibroma is clearly demarcated from the myocardium, but interdigitations with the surrounding myocardium can be seen on microscopy (Figures 9 and 10). Calcification of the central portion of the tumour is pathognomonic for fibroma reflecting poor blood supply to the mass [29].

Cardiac fibromas usually remain dormant and spontaneous regression rarely occurs, therefore total surgical resection is normally recommended. Large tumours can be resected subtotally. If progressive loss of working myocardial fibres continues and cardiac failure does not resolve following tumour resection, heart transplantation may be considered [30,31].

### Teratoma

**Figure 11:**
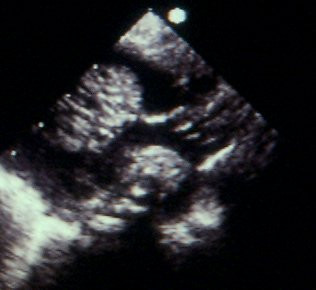
Cystic teratoma (*) attached to the aortic root (Ao).

Cardiac teratoma is a rare tumour of the heart and pericardium [32]. Teratomas are the second most common tumour in the fetus and neonate after rhabdomyoma [33-36]. Most commonly, these tumours are detected in the pericardial cavity attached to the pulmonary artery and aorta [37] (Figure 11). The tumour size within the heart varies from 2 to 9 cm in diameter, and intrapericardial tumours as large as 15 cm have been reported [37,38]. Intracardiac tumours arise from the atrial or ventricular wall as nodular masses protruding into the cardiac chambers. Cardiac and pericardial teratomas are easily detected in the fetus and neonate by two-dimensional echocardiography as heterogeneous and encapsulated cystic masses.

**Figure 12:**
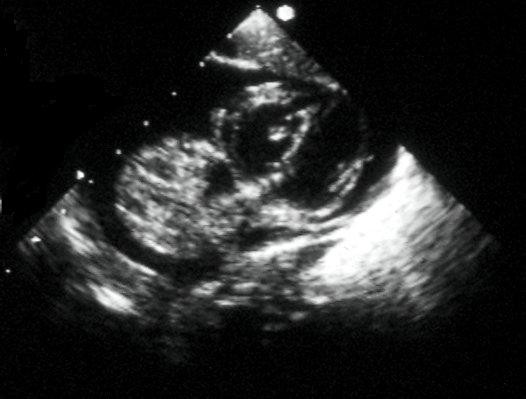
Pericardial effusion (*) and cystic teratoma (♥) in a child.

The main clinical findings in the foetus or neonate relate to the mass effect of the tumour and to the accumulation of fluid in the pericardial space (Figure 12). Fetal hydrops and stillbirth may occur. Respiratory distress, cyanosis, and congestive heart failure are predominant signs in the neonate. Pericardial effusion may lead to cardiac compression and tamponade. The pericardial effusion is usually serous and contains small numbers of mesothelial cells [39].

**Figure 13:**
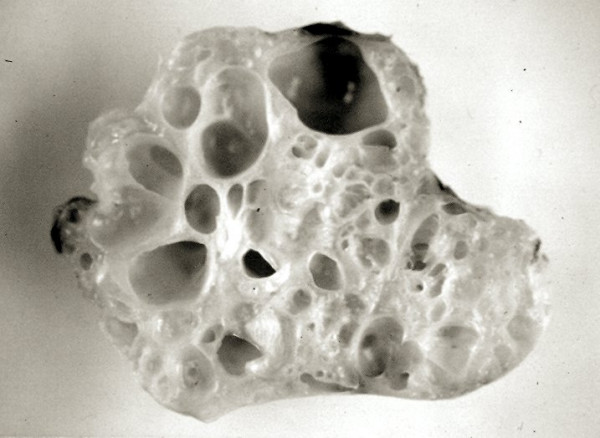
Typical cystic and multi-lobular appearance of a teratoma excised from heart.

Macroscopically teratomas have a typical cystic and multilobulated appearance (Figure 13).

**Figure 14:**
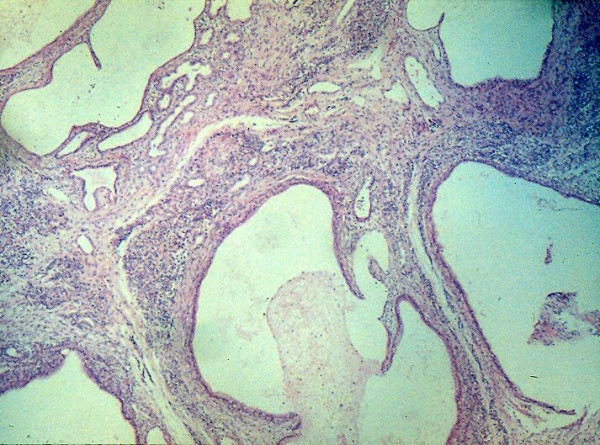
Histology of a teratoma showing multiple immature elements in it.

Teratomas histologically contain multiple immature elements including epithelium, neuroglial tissue, thyroid, pancreas, smooth and skeletal muscle, cartilage and bone (Figure 14).

Although teratomas have been considered to be benign, tumour recurrence after resection or malignant differentiation has been reported. Most intrapericardial teratomas can be easily dissected off from the great vessels; on the contrary, surgical removal of intracardiac ones is technically more difficult [40].

### Myxoma

Myxoma is the most common primary cardiac tumour in adults (65%) with particular predilection to females [41]. The left atrium is the most common location (90%) but they can be seen on the right atrium as well [5]. In 90% of cases the myxoma is solitary. Myxomas usually do not cause cardiac murmurs, but more often they present with breathlessness, syncope, embolus, congestive heart failure, arrhythmias and constitutional symptoms. As the presenting symptoms may not suggest a cardiac cause, myxomas may present late in the disease and at this stage cardiac signs may then also ensue. On occasions, left-sided myxomas may cause diastolic murmurs or added sounds ("diastolic plop") due to inflow obstruction. Right-sided myxomas are rare and they may lead to tricuspid or pulmonary artery obstruction or pulmonary embolism.

Familial occurrence of myxomas has been reported, usually seen in younger patients [42]. These myxomas are associated with multiple endocrine syndromes including LAMB (lentigines, atrial myxoma, mucocutaneous myxoma, and blue naevi) and NAME (naevi, atrial myxoma, myxoid neurofibromata, and ephelides). Familial tumours are predominantly multiple but the sporadic cases are mostly solitary. Screening of first-degree relatives cases of multiple myxomas should therefore be considered.

Macroscopically, myxomas are pedunculated with a short, broad-based attachment to the atrial wall. They are gelatinous in consistency. The echocardiographic appearance of a myxoma is highly suggestive of the diagnosis: it has a pedicle, with irregular, non-homogenous small lucencies or calcifications on it and the location is characteristically to the atrial septum, around foramen ovale [43-45]. In contrast, an intracardiac thrombus is usually homogenous in appearance.

The treatment of choice is complete surgical excision of the myxoma with removal of substantial portion of healthy adjacent endocardial tissue. If the surrounding tissue along with the myxoma is not resected completely, the tumour is likely to recur. Prognosis following surgery is quite good, albeit with a recurrence rate of up to a 5% if there has been inadequate resection, intraoperative implantation, tumour embolisation, or unrecognised multiple lesions [46,47]. Survival after surgery has been reported to be excellent with no late deaths attributable to surgery or to myxoma recurrence during 16 years of follow up [48].

### Haemangioma

Haemangioma is an exceedingly rare tumour and can be located anywhere within the cardiac layers with slight predilection to the ventricular septum and right atrium [49,50]. Macroscopically, haemangiomas are subendocardial nodules measuring 2–4 cm in diameter. Microscopically, they may have capillary, cavernous, intramuscular or haemangioendotheliomatous features. The presence of multiple haemangiomas in the skin should raise suspicion of a cardiac haemangioma in the neonate [51].

Haemangiomas are benign tumours and undergo spontaneous regression with a good prognosis. However, their clinical course may be unfavourable in infants due to high-output cardiac failure, haemorrhage from ruptured vessels, and thrombocytopaenia. Ventricular tachycardia and cardiac tamponade are not uncommon [51]. Complete surgical excision may be difficult because of the vascular nature of the tumour [52].

### Histiocytoid nodule – oncocytic cardiomyopathy – Purkinje cell tumour

These hamartomatous masses differ distinctly from rhabdomyomas. They are mostly found in infants and young children presenting with refractory arrhythmias or sudden unexpected death. They have a predilection to the female sex. These masses have been described with a variety of names such as infantile histiocytic cardiomyopathy, oncocytic cardiomyopathy, histiocytoid cardiomyopathy, Purkinje cell tumour, focal lipid cardiomyopathy, and idiopathic infantile cardiomyopathy [53,54].

The most common clinical presentation is refractory arrhythmias such as ventricular fibrillation, ventricular tachycardia, and junctional tachycardia [55-58]. Premature ventricular contractions, supraventricular tachycardia, and partial or complete atrioventricular block can also be seen. Oncocytic cardiomyopathy is an important cause of sudden unexpected death in infancy [59].

Macroscopically, these masses present as focal yellowish nodules or yellow areas of discoloration composed of vacuolated histiocyte-like cells within the myocardium. In up to one-third of patients the tumours may be associated with cardiac or extracardiac anomalies such as atrial and ventricular septal defects, hypoplastic left heart syndrome, cleft palate, and anomalies of the eyes, skin and central nervous system [59,60]. Their size varies from 1 to 2 mm in diameter. The most common locations are conduction system and the left ventricle [58].

**Figure 15:**
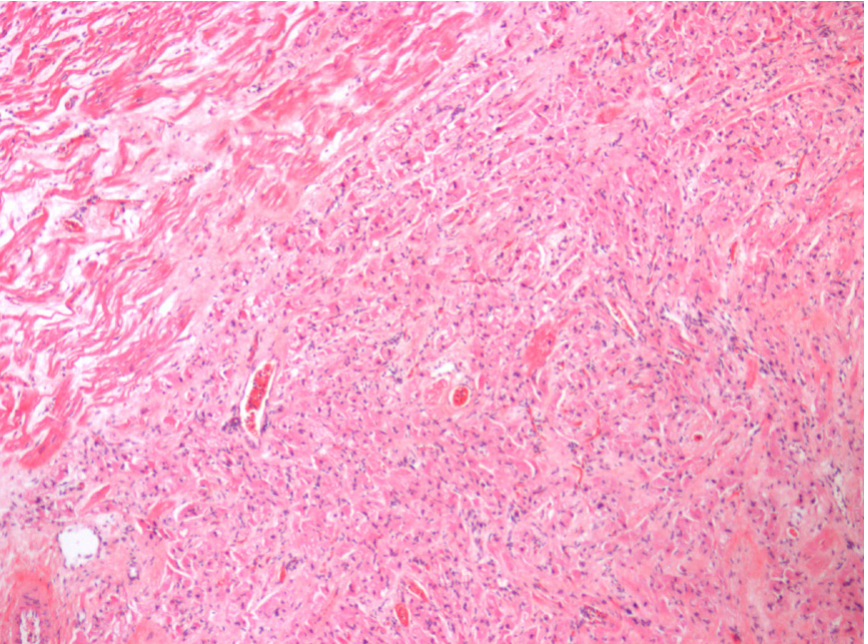
Microscopically histiocytoid nodules contain large oval cardiac myocytes with coarse granular pale cytoplasm.

**Figure 16:**
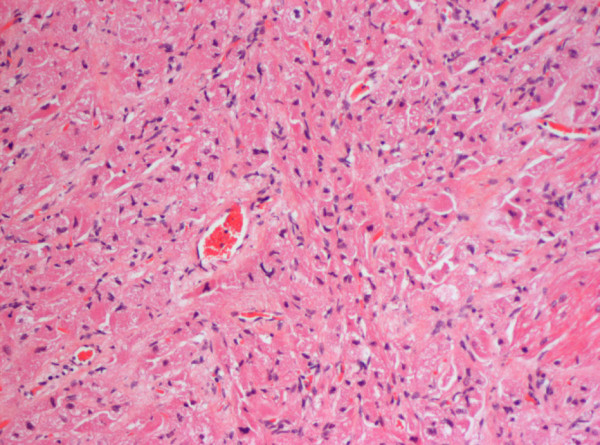
High power microscopy showing enlarged, granular-appearing myocytes.

Microscopically, these masses contain large oval cardiac myocytes with a coarse granular pale cytoplasm (Figure 15). The cytoplasm is filled with bizarre looking mitochondria. The term oncocytic cardiomyopathy describes the process of the granules (mitochondria) replacing the working myofibrils (Figure 16). Infants with oncocytic cardiomyopathy may show similar oncocytic cells in other organs including the trachea, adrenal, thyroid, anterior pituitary, and salivary glands [60,61].

Echocardiography may reveal nodular deposits on the ventricular endocardium or valves [62]. If left untreated, oncocytic cardiomyopathy may have a fatal course in infants with multiple unresectable lesions. Some patients may benefit from electrophysiological studies with mapping and surgical excision [57,63].

### Lipoma

Cardiac lipomas are rare lesions and occur exclusively in adults. They are encapsulated subepicardial masses. They are commonly silent but rarely may cause symptoms including arrhythmias and atrioventricular block [64,65].

## Malignant cardiac tumours

### Primary malignant cardiac tumours

Up to a quarter of all cardiac tumours may exhibit some features of malignancy. Ninety five percent of these primary malignancies are sarcomas [66], the other 5% are lymphomas. Sarcomas are more common in adults and most commonly located in the right atrium. These mesenchymal tumours show various morphologies. The clinical course is usually aggressive with extensive local infiltration, intracavity obstruction and death.

### Angiosarcoma

Angiosarcomas are the most common primary cardiac malignancy and more common in male sex. Eighty percent of these tumours originate in the right atrium or pericardium [66,67]. Echocardiography shows a broad based atrial mass close to the inferior vena cava with frequent epicardial, endocardial or intracavity extensions. Local pulmonary, pleural and mediastinal metastases are frequent. Clinical findings include right-sided heart failure, pericardial disease, pleuritic chest pain, dyspnoea, and pericardial effusion. Some patients (10%) may present with nonspecific symptoms such as fever, weight loss, and malaise. The outlook is poor, and most patients die shortly after the onset of symptoms.

### Rhabdomyosarcoma

Rhabdomyosarcomas are the second most common primary malignancy of the heart. Their origin is of striated muscle. These malignancies are most common in adult males and can occur in any heart chamber. The most common presenting symptoms are nonspecific including fever, anorexia, malaise, and weight loss. The symptoms associated with pericardial disease, pleural effusion, and embolic phenomena are also common. As compared to angiosarcoma, diffuse pericardial involvement is not common and the tumour rarely invades beyond the parietal pericardium. The prognosis is poor [66,69].

### Fibrosarcoma

Fibrosarcomas are mesenchymal tumours with fibroblastic origin. These tumours can be seen within the left or right heart chambers [66,69]. At postmortem they are usually found in multiple intracardiac sites as firm, greyish-white small nodules. The clinical findings include heart murmurs, nonspecific ECG changes, chest pain, fever, and malaise. They may spread to surrounding structures [25]. The outlook is poor with death ensuing within a year of diagnosis.

### Lymphoma

Primary lymphoma of the heart is rare and involves only the heart or the pericardium [70]. These tumours man be seen in various cardiac sites without any specific predilection. They may present with congestive heart failure, cardiomegaly, or pericardial effusion and may be seen as part of the acquired immunodeficiency syndrome [71]. Surgical resection and radiotherapy offer limited success.

### Intrapericardial phaeochromocytoma

These tumours are soft, vascular and originate from autonomic paraganglia. They may be found within the atrium, in the atrial septum, in coronary, pulmonary, or aortic locations. If the tumour can be resected, the prognosis is favourable [72,73].

### Secondary malignant (metastatic) cardiac tumours

The incidence of cardiac metastases with malignant tumours is estimated to be approximately 1% and 20 times higher than primary malignancies of the heart [4,74]. The most common cardiac metastases originate from lung tumours in men and breast tumours in women. Metastatic tumours are mostly located in the epicardial surface of the heart.

Macroscopically carcinomatous metastatic tumours are multiple small, discrete, and firm nodules. Carcinomas are more frequent than sarcomatous infiltrations.

Melanoma shows a special affinity to spread to heart with equal distribution to all four chambers [75]. Leukemias and lymphomas may cause intramyocardial infiltration, haemorrhagic pericardial effusion, but occasionally they can remain asymptomatic [66,70,71,76].

The majority of metastatic tumours remain silent but some may present with arrhythmias, cardiac failure, or pericardial effusion.

## Diagnostic methods

Echocardiography, Computing Tomography (CT) and MRI of the heart are the main non-invasive diagnostic tools. Open surgical or endomyocardial biopsy is only utilised to reveal the histology of the lesion before surgical resection. A thorough metastatic check should be carried out with CT or MRI imaging before removal of malignant cardiac tumours. A bone marrow biopsy or bone scan may be necessary.

## Treatment

### Resection

Myxomas should be excised completely along with a small rim of surrounding healthy myocardium. In view of their potential for embolisation or valvular obstruction, early resection of myxomas is recommended.

Surgical resection of primary cardiac tumours should only be considered to relieve symptoms and mechanical obstruction to blood flow. The outcome of surgical resection in symptomatic, non-myxomatous benign cardiac tumours is favourable [77,78].

Primary cardiac malignancies may benefit from palliative surgery but this approach cannot be recommended for patients with metastatic cardiac tumours. Surgery, chemotherapy and radiotherapy may prolong survival. The prognosis for malignant primary cardiac tumours is generally extremely poor.

### Transplantation

If the resection of the tumour is not achievable in severely symptomatic patients, orthotopic cardiac transplantation may be considered. This treatment modality is only an option for patients with unresectable cardiac tumours with no evidence of metastatic involvement of the heart.

## Competing interests

The author(s) declare that they have no competing interests.

## Authors' contributions

OU is the principal author and editor of this article. He was commissioned to write the review. He has designed, written, reviewed the article and has given final approval of the version to be published. OU also provided the figures from his own collection. DGW revised the final manuscript and helped with the organisation of references. DGW, GMV, JMP and JVDG have been involved in revising the article critically for important intellectual content. GMV has also provided figures 6 and 11 from his collection. All authors read and approved the manuscript.
